# Effectiveness of Nivolumab in Second-Line and Later in Patients with Advanced Non-Small Cell Lung Cancer in Real-Life Practice in France and Germany: Analysis of the ESME-AMLC and CRISP Cohorts

**DOI:** 10.3390/cancers14246148

**Published:** 2022-12-13

**Authors:** Christos Chouaid, Michael Thomas, Didier Debieuvre, Isabelle Durand-Zaleski, Stefan Zacharias, Lise Bosquet, Annika Groth, Annette Fleitz, Alan Calleja, Sonya Patel, Laure Lacoin, Melinda J. Daumont, John R. Penrod, Robert Carroll, Daniela Waldenberger, François-Emery Cotté, Clarisse Audigier-Valette, Frank Griesinger

**Affiliations:** 1 Pneumology Unit, Centre Hospitalier Intercommunal de Créteil, 40 Avenue de Verdun, 94000 Créteil, France; 2 Translational Lung Research Center Heidelberg (TLRC-H), Member of the German Center for Lung Research (DZL), 69126 Heidelberg, Germany; 3 Thoraxklinik and National Center for Tumor Diseases at Heidelberg University Hospital, Röntgenstr. 1, 69126 Heidelberg, Germany; 4 Chest Disease Department, GHRMSA–Emile Muller Hospital, 68070 Mulhouse, France; 5 AP-HP Health Economics Research Unit, Hotel Dieu Hospital, INSERM UMR 1153 CRESS, Clinical Epidemiology (Methods) Research Team, 75004 Paris, France; 6 Department of Biostatistics, iOMEDICO, Ellen-Gottlieb-Straße 19, 79106 Freiburg, Germany; 7 Health Data and Partnerships Department, Unicancer, 101 Rue de Tolbiac, CEDEX 13, 75654 Paris, France; 8 AIO-Studien-gGmbH, Kuno-Fischer-Str. 8, 14057 Berlin, Germany; 9 Clinical Epidemiology and Health Economics, iOMEDICO, Ellen-Gottlieb-Straße 19, 79106 Freiburg, Germany; 10 Real World Solutions, IQVIA, The Point, 37 North Wharf Road, London W2 1AF, UK; 11 Epi-Fit, 261 Rue Mandron, 33000 Bordeaux, France; 12 Worldwide Health Economics & Outcomes Research, Bristol Myers Squibb, Avenue de Finlande 4, 1420 Braine-L’Alleud, Belgium; 13 Worldwide Health Economics & Outcomes Research, Bristol Myers Squibb, 3551 Lawrenceville, Princeton Rd, Princeton, NJ 002627, USA; 14 Centre for Observational Research & Data Sciences, Bristol Myers Squibb, Uxbridge Business Park, Sanderson Road, Uxbridge UB8 1DH, UK; 15 Medical Oncology, Bristol Myers Squibb GmbH & Co. KGaA, Arnulfstraße 29, 80636 Munich, Germany; 16 Health Economics & Outcomes Research, Bristol Myers Squibb, 3 Rue Joseph Monnier, 92500 Rueil-Malmaison, France; 17 Department Thoracic Oncology, Centre Hospitalier Toulon Sainte Musse, 54 Rue Henri Sainte Claire Deville, 83056 Toulon, France; 18 Department of Haematology & Oncology, University Department Internal Medicine-Oncology, Pius-Hospital, University Medicine Oldenburg, Georgstr. 12, 26121 Oldenburg, Germany

**Keywords:** nivolumab, non-small cell lung cancer, immune checkpoint inhibitor, clinical practice, I-O Optimise, real-world evidence, immunotherapy, programmed death-ligand 1

## Abstract

**Simple Summary:**

There is a need to better understand the effectiveness of new treatments, such as the recently approved nivolumab, in patients with locally advanced or metastatic non-small cell lung cancer in clinical practice. This study aims to report the characteristics and outcomes of 2784 patients with locally advanced or metastatic non-small cell lung cancer receiving nivolumab in second-line or later in France (ESME-AMLC) and Germany (CRISP) between 2015 and 2020. Two-year survival rates were 26.7% in patients with tumors with squamous histology and 32.8% in patients with non-squamous/others histologies in ESME-AMLC, and 20.9% and 18.9%, respectively, in CRISP. Poorer performance score and shorter duration from the previous line of therapy initiation were significantly associated with shorter treatment duration with nivolumab and overall survival. These real-world data provide insight into the characteristics of patients receiving nivolumab in France and Germany and confirm the efficacy of nivolumab previously observed in clinical trials.

**Abstract:**

This study reports characteristics and outcomes in patients with locally advanced or metastatic non-small cell lung cancer (aNSCLC) receiving nivolumab in second-line or later (2L+) in France and Germany between 2015 and 2020. Patients with aNSCLC (stage IIIB–C/IV) receiving nivolumab in 2L+ were included from the retrospective Epidemiological Strategy and Medical Economics of Advanced and Metastatic Lung Cancer cohort (ESME-AMLC, France; 2015–2019) and Clinical Research platform Into molecular testing, treatment and outcome of non-Small cell lung carcinoma Patients (CRISP, Germany; 2016–2020). Overall, 2262 ESME-AMLC and 522 CRISP patients were included. Median treatment duration (95% confidence intervals) was 2.8 months (2.5–3.2) in squamous and 2.5 months (2.3–2.8) in non-squamous/others patients in ESME-AMLC, and 2.3 months (1.4–3.1) and 2.3 months (2.0–2.8), respectively in CRISP. One-year and two-year overall survival (OS) were 47.2% and 26.7% in squamous and 50.8% and 32.8% in non-squamous/others patients in ESME-AMLC, and 43.1% and 20.9%, and 37.7% and 18.9%, respectively in CRISP. Poorer performance score and shorter time from start of previous line of therapy initiation were significantly associated with shorter treatment duration and OS. This study confirms, in real-world clinical databases, the efficacy of nivolumab previously observed in clinical trials.

## 1. Introduction

In 2020, lung cancers were the leading cause of cancer-related deaths in France and Germany, accounting for 37,095 and 50,282 deaths, respectively [1,2]. Non-small cell lung cancers (NSCLCs), including squamous cell carcinomas, large cell carcinomas and adenocarcinomas, account for 85−90% of lung cancers. Of these, squamous NSCLCs account for approximately one quarter of lung cancers [3].

The European Society for Medical Oncology (ESMO) guidelines list immunotherapy and, in particular, immune checkpoint inhibitors (ICIs) as having a vital role in the treatment of NSCLC and recommend immunotherapy as part of systemic anti-cancer therapy (SACT; any chemotherapy, immunotherapy and targeted biological therapy) for patients with non-oncogene-driven locally advanced or metastatic non-small cell lung cancer (aNSCLC, stage IIIB–C/IV) [4].

Nivolumab was the first ICI approved for aNSCLC. Nivolumab is a fully human immunoglobulin (Ig) G4 antibody belonging to a class of ICIs targeting interactions between the immune checkpoint receptor programmed death 1 (PD-1) and its ligand, programmed death-ligand 1 (PD-L1) [4]. Levels of PD-L1 are often increased in patients with NSCLC. Tumor expression of PD-L1 suppresses anti-tumor immune responses by binding to PD-1 expressing T-cells and B-cells, preventing immune cell activation and resultant tumor detection and destruction [5,6]. Nivolumab acts to inhibit PD-1/PD-L1 interactions to enable recognition of the tumor by circulating immune cells. Once recognized, immune cells act to destroy tumor cells, thus delaying tumor growth [5].

Nivolumab has demonstrated greater median overall survival (OS), 1-year survival rates, 1-year progression-free survival (PFS), higher response rates and an improved safety profile compared with docetaxel in patients with aNSCLC, for both squamous (SQ) and non-squamous (NSQ) histologies, in randomized, open-label, phase 3 clinical trials [7,8]. Further analysis has shown that this survival benefit was maintained over 2 and 5 years of follow-up [9,10]. Nivolumab efficacy was also correlated with tumor PD-L1 levels in patients with NSQ aNSCLC, but not SQ aNSCLC in these trials [7,8,10].

Nivolumab monotherapy is approved as a second-line (2L) therapy for patients with aNSCLC following chemotherapy and has more recently been approved for use in combination with ipilimumab and two cycles of platinum-based chemotherapy as first-line (1L) treatment in patients with aNSCLC without sensitizing epidermal growth factor receptor (*EGFR*) or anaplastic lymphoma kinase (*ALK*) mutations [11]. In France, nivolumab became available as a 2L therapy for SQ and NSQ aNSCLC through the early access Temporary Authorization for Use (ATU) from January 2015 and was then reimbursed for 2L SQ NSCLC in December 2016 and NSQ in March 2017. Whereas in Germany, as new therapies are reimbursed directly after European Medicines Agency (EMA) approval, nivolumab has been reimbursed for 2L therapy for SQ and NSQ NSCLC from July 2015 and April 2016, respectively.

Following approval, the real-world use of nivolumab in clinical practice and outcomes in patients receiving nivolumab is of clinical interest. In combination with data from randomized clinical trials, real-world studies can help to inform future decisions on the use of ICIs, including nivolumab, in clinical practice. The I-O Optimise programme is a multinational, observational research platform utilizing real-world data sources to generate insights on the treatment and outcomes of patients with lung cancer [12]. A recent study, published as part of the I-O Optimise initiative, described the evolution of the use of ICIs in clinical practice in France and Germany between 2015 and 2020 using two large clinical databases, the Epidemiological Strategy and Medical Economics of Advanced and Metastatic Lung Cancer (ESME-AMLC) data platform in France and the Clinical Research platform Into molecular testing, treatment and outcome of non-Small cell lung carcinoma Patients (CRISP) data source in Germany, respectively [13]. The present study was conducted to evaluate the effectiveness of nivolumab in clinical practice in patients with aNSCLC receiving nivolumab treatment in 2L or later (2L+) using the same clinical databases. The hypothesis of this study was that the efficacy of nivolumab in the real world will reflect results observed in clinical trials. This study also aimed to identify factors associated with duration of treatment and OS of these patients.

This study showed that, overall, the clinical outcomes of patients with aNSCLC treated with nivolumab in the real-world setting in the ESME-AMLC and CRISP databases were consistent between countries and with those previously reported in randomized clinical trials (RCTs).

## 2. Materials and Methods

This study is an observational cohort study, based on two existing data sources: the ESME-AMLC data platform in France, and the CRISP data source in Germany.

### 2.1. ESME-AMLC

The ESME-AMLC research program is an academic real-world data platform retrospectively collecting and centralizing comprehensive data on cancer management from a network of academic and non-academic health facilities (private non-profit comprehensive cancer centers, and university or general hospitals). The sites within the network are selected to be representative of the French healthcare system for the treatment of advanced and metastatic lung cancer. At the time of the analysis, 30 sites were contributing to the ESME-AMLC database, although there are now 39 in place.

The dataset (NCT03848052) was authorized by the French data protection authority in 2017 and focuses on adult patients with aNSCLC (stage IIIB/IV) who were diagnosed or initiated treatment from 2015 onwards. Data are compiled from patient’s electronic medical records, inpatient hospitalization records and pharmacy records, and employs rigorous screening procedures. Data from multiple data sources within each center (e.g., French computerized medical information system, pharmacy records, other databases or search engines) are used to comprehensively identify all patients that meet the selection criteria. Data were retrospectively collected from medical records using a well-structured electronic data collection tool approach by trained technicians on-site.

### 2.2. CRISP (AIO-TRK-0315)

CRISP (NCT02622581) is an open, non-interventional, prospective, multicenter clinical research platform, that collects data from more than 170 cancer sites (certified lung cancer centers, comprehensive cancer centers, university and non-university hospitals, and office-based oncology practices) and includes, among other cohorts, adult patients with aNSCLC at the start of 1L systemic therapy. Inclusion criteria for patients in the NSCLC advanced stage cohort are (i) Stage IIIB (if the patient is ineligible for curative surgery and/or radiochemotherapy), Stage IIIC (if the patient is ineligible for curative surgery and/or radiochemotherapy) or Stage IV (histologically confirmed NSCLC); (ii) signed informed consent no later than 4 weeks after the start of 1L treatment; and (iii) ability to understand and complete the patient-reported outcome assessment. The first patient was recruited in December 2015 and the current dataset represents approximately 3–6% of the aNSCLC population in Germany.

### 2.3. Study Population

Among patients diagnosed between 2015 and 2018 in the ESME-AMLC and CRISP data sources, the present study included all adult patients receiving nivolumab in 2L+ for aNSCLC. Patients with concomitant cancers were excluded (i.e., diagnosis of another cancer within 5 years prior to aNSCLC diagnosis or any ongoing SACT regimen at the time of aNSCLC diagnosis). Patients who received nivolumab as part of a clinical trial were also excluded. Patients were followed up from the start of nivolumab to the date of the last patient status/last contact of the patient with the center prior to the end of the study period, known exit from the data source or death.

### 2.4. Study Period

The study period for ESME-AMLC was from January 2015 to August 2019 (date of the last data extraction). The study period for CRISP was from January 2016 to June 2020.

### 2.5. Analyses

Patient and clinical characteristics were summarized using descriptive statistics. Analyses were stratified into two histological categories: SQ histology, and NSQ and all others (including undifferentiated carcinoma) (NSQ/others).

OS, PFS and treatment duration were estimated using survival methodology, depicted graphically by Kaplan–Meier curves. For ESME-AMLC, a sensitivity analysis was carried out for OS to evaluate the maximum bias that could be introduced by the underreporting of deaths in the patients’ medical records. In this analysis, all censored patients with a last contact ≥ 12 months prior to the cut-off date for the study were considered as having died.

Treatment response was collected in CRISP only and was defined by the best clinical response according to the physician’s assessment as per local site standard, which was the best tumor response observed within each line of therapy (LoT). Categories were complete response, partial response, stable disease and progressive disease.

Reasons for treatment discontinuation were defined as progression, toxicity, patient’s choice (in ESME-AMLC only), doctor’s choice/protocol driven choice (in ESME-AMLC only), according to guidelines/protocol (in CRISP only) and ‘other’.

Factors associated with nivolumab treatment duration and factors associated with OS in patients treated with nivolumab 2L+ were assessed using univariate Cox proportional hazards regression modeling to provide crude hazard ratios (HRs), 95% confidence intervals (CIs) and a *p*-value for each variable. A fully adjusted multivariate Cox model was carried out including all preselected variables with *p* < 0.20 in the univariate analyses as well as the following a priori variables: age, sex and Eastern Cooperative Oncology Group (ECOG) performance status, providing adjusted HRs, 95% CIs and a *p*-value for each variable. Each data source was analyzed separately, applying consistent variable definitions and analytic methods. Differences in data collection and analysis techniques have the potential to impact research findings and their interpretation; therefore, instances of heterogeneity across the two data sources are highlighted and discussed.

## 3. Results

### 3.1. Patient Characteristics

During the study period, 2262 patients in ESME-AMLC and 522 patients in CRISP received nivolumab in 2L+ for the treatment of aNSCLC. The characteristics of these patients at the time of 2L+ nivolumab treatment start are presented in Table 1, overall and by histological subgroups.

Patients with SQ histology represented one fourth of the patients treated with nivolumab in both data sources. Patients treated with nivolumab in CRISP were older than those in ESME-AMLC. Median age at initiation of nivolumab was 63.5 years and 66 years in ESME-AMLC and CRISP, respectively. The proportion of patients over 75 years old was 11.6% in ESME-AMLC and 24.1% in CRISP. Median age was higher in SQ patients compared with NSQ/others patients in both ESME-AMLC (66.1 years vs. 62.5 years) and CRISP (69.0 years vs. 64.0 years). Of the nivolumab-treated patients in ESME-AMLC and CRISP, 90.7% and 97.1% had at least one metastasis at the time of nivolumab start, respectively. The proportion with brain metastases in ESME-AMLC was 19.6% among patients with SQ histology and 39.8% among NSQ/others. The proportion with brain metastases in CRISP was 12.0% among SQ patients and 28.8% among NSQ/others patients. The proportion of patients with *EGFR, ALK* or *ROS* (c-ros oncogene) mutations was low in both ESME-AMLC (3.5%, 0.8% and 0.6%, respectively) and CRISP (4.6%, 1.9% and 1.5%, respectively).

Overall, 30.0% of the ESME-AMLC cohort and 59.4% of the CRISP cohort received at least one PD-L1 test prior to starting nivolumab. A decrease in the proportion of patients not tested for PD-L1 prior to nivolumab use was observed over the study period (from 90.3% in 2015 to 23.1% in 2019 in ESME-AMLC, and from 71.4% in 2016 to 20.4% in 2019 in CRISP). This was mostly associated with an increase in the proportion of PD-L1-negative patients treated with nivolumab (from 3.2% to 43.1% in ESME-AMLC and from ≤10% to 58.4% in CRISP) (Table S1).

The proportion of patients receiving nivolumab in 2L was 67.0% in ESME-AMLC and 83.0% in CRISP. The proportion of patients receiving nivolumab in 3L or later was 33.0% in ESME-AMLC and 17.1% in CRISP. The median time from initial diagnosis to nivolumab start was 9.6 months (Q1–Q3; 6.2–15.9) in ESME-AMLC and 7.8 months (Q1–Q3; 5.2–11.8) in CRISP. Most of the patients had received at least one platinum-based chemotherapy prior to nivolumab (90.7% in ESME-AMLC and 94.3% in CRISP). The median time from the start of the previous LoT until the start of nivolumab was 5.5 months in ESME-AMLC and 5.4 months in CRISP, and the last LoT regimen received prior to nivolumab was a platinum-based chemotherapy for 70.3% and 84.1% of patients, respectively.

### 3.2. Treatment Response, Treatment Duration and Reasons for Discontinuation

Best treatment response, available in the CRISP database only, are presented overall, by histology and by PD-L1 status in Table 2. Of patients who were treated with 2L+ nivolumab, 11.1% achieved treatment response (complete or partial), 20.5% remained stable, 31.4% progressed and 37.0% had an unknown response. Treatment response was similar between histological subgroups. PD-L1 data should be interpreted with caution due to a high number of unknown treatment responses.

**Figure 1:**
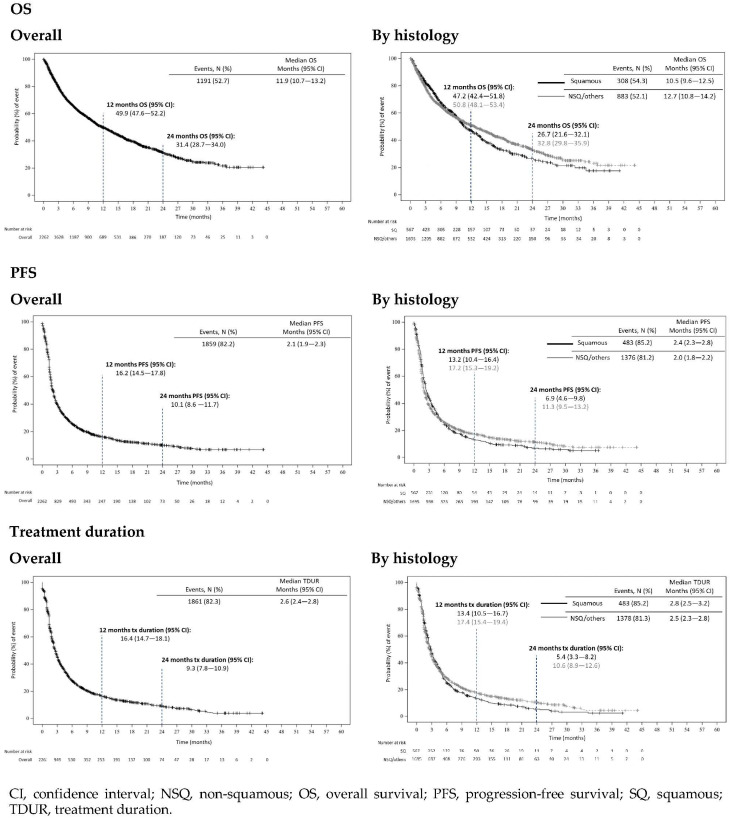
OS, PFS and treatment duration by histology in patients treated with nivolumab in ESME-AMLC (index date = nivolumab start date).

In ESME-AMLC, median (95% CI) nivolumab treatment duration was 2.8 months (2.5–3.2) in SQ patients and 2.5 months (2.3–2.8) in NSQ/others. At 12 months after nivolumab start, 13.4% (10.5–16.7) and 17.4% (15.4–19.4) were still on treatment, and 5.4% (3.3–8.1) and 10.6% (8.9–12.6) were still on treatment at 24 months in SQ and NSQ/others patients, respectively (Figure 1). The main reason for nivolumab discontinuation was progression (57.7%), although 8.2% discontinued due to toxicity (Table 3).

**Figure 2:**
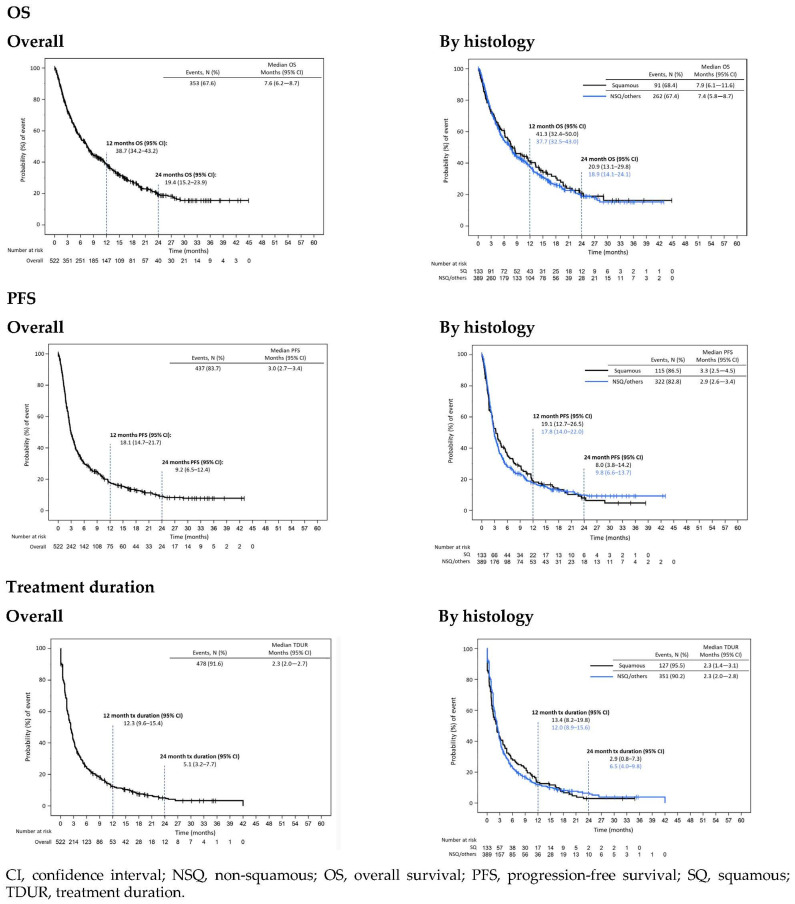
OS, PFS and treatment duration by histology in patients treated with nivolumab in CRISP (index date = nivolumab start date).

In CRISP, median (95% CI) nivolumab treatment duration was 2.3 months (1.4–3.1) in SQ patients and 2.3 months (2.0–2.8) in NSQ/others. At 12 months after nivolumab start, 13.4% (8.2–19.8) and 12.0% (8.9–15.6) were still on treatment and 2.9% (0.8–7.3) at 6.5% (4.0–9.8) were still on treatment at 24 months in SQ and NSQ/others patients, respectively (Figure 2). The main reason for nivolumab discontinuation was also progression (51.9%), with 5.4% discontinuing due to toxicity (Table 3).

### 3.3. OS and PFS

#### 3.3.1. ESME-AMLC

After a median follow-up time of 9.9 months (Q1–Q3, 4.2–18.7), 47.3% of the patients were censored in ESME-AMLC. Median OS (95% CI) in ESME-AMLC from start of 2L+ nivolumab treatment was 11.9 months (10.7–13.2); median OS was 10.5 months (9.6–12.5) in SQ patients and 12.6 months (10.8–14.2) in NSQ/others. One-year and two-year OS were 47.2% (42.4–51.8) and 26.7% (21.6–32.1) in SQ patients, and 50.8% (48.1–53.4) and 32.8% (29.8–35.9) in NSQ/others (Figure 1). Sensitivity analyses for OS are presented in Figure S1.

Median PFS (95% CI) from start of 2L+ nivolumab treatment was 2.1 months (1.9–2.3); median PFS was 2.4 months (2.3–2.8) in SQ patients and 2.0 months (1.8–2.2) in NSQ/others. One-year and two-year PFS were 13.2% (10.4–16.4) and 6.9% (4.6–9.8) in SQ patients, and 17.2% (15.3–19.2) and 11.3% (9.5–13.2) in NSQ/others (Figure 1).

#### 3.3.2. CRISP

After a median follow-up time of 4.3 months (Q1–Q3, 0.5–12.4), 32.4% of the patients were censored in CRISP. Median OS (95% CI) from the start of 2L+ nivolumab treatment was 7.6 months (6.2–8.7); median OS was 7.9 months (6.1–11.6) in SQ patients and 7.4 months (5.8–8.7) in NSQ/others. One-year and two-year OS were 43.1% (32.4–50.0) and 20.9% (13.1–29.8) in SQ patients, and 37.7% (32.5–43.0) and 18.9% (14.1–24.1) in NSQ/others (Figure 2).

Median PFS (95% CI) from start of 2L+ nivolumab treatment was 3.0 months (2.7–3.4); median PFS was 3.3 months (2.5–4.5) in SQ patients and 2.9 months (2.6–3.4) in NSQ/others. One-year and two-year PFS were 19.1% (12.7–26.5) and 8.0% (3.8–14.2) in SQ patients, and 17.8% (14.0–22.0) and 9.8% (6.6–13.7) in NSQ/others (Figure 2).

### 3.4. Factors Associated with Treatment Duration and OS

Poor functioning, measured by the ECOG performance scale, was negatively associated with nivolumab treatment duration and OS in both data sources, while a longer time from start of previous LoT until starting nivolumab was positively associated with treatment duration and OS (Table 4 and Table 5; Tables S2–S5).

Negative PD-L1 expression (i.e., PD-L1 < 1%) was also found to be negatively associated with treatment duration (*p* = 0.03 in EMSE-AMLC and *p* = 0.06 in CRISP), and a significant negative association with OS was also observed in CRISP (*p* < 0.01).

The absence of any distant metastasis at time of nivolumab start was positively associated with treatment duration and OS compared with the presence of brain metastases in ESME-AMLC (*p* = 0.03 and *p* = 0.05). A similar trend was observed in CRISP associated with OS, although this was not statistically significant (*p* = 0.17) and potentially due to the limited number of patients without any metastases in CRISP (n = 15). In ESME-ALMC, the presence of *EGFR*, *ALK* or *ROS* mutations was negatively associated with treatment duration (*p* < 0.01), and there was a trend for a positive association with OS in current and former smokers compared with never smokers (*p* = 0.07).

## 4. Discussion

Nivolumab was the first ICI approved for the treatment of aNSCLC. RCTs have demonstrated improved survival outcomes with nivolumab compared with docetaxel in patients with aNSCLC after prior systemic treatment [7,8,9,10]. This real-world study utilized two of the largest lung cancer databases in Europe (ESME-AMLC in France and CRISP in Germany) to evaluate patient characteristics, treatment duration, PFS and OS outcomes in patients who received nivolumab for aNSCLC after prior systemic treatment between 2015 and 2020.

Due to this real-world nature, the demographics of patients receiving nivolumab as 2L+ therapy in ESME-AMLC and CRISP were broader than those included in the previous clinical trials of nivolumab, CheckMate 017 and CheckMate 057 [7,8]. Patients tended to be older in the present study (11.6% and 24.1% aged ≥75 years in ESME-AMLC and CRISP, respectively, versus 8% and 7% aged ≥75 years in CheckMate 017 and CheckMate 057, respectively) and had a lower performance status (patients with an ECOG score of ≥2 were not included in pivotal nivolumab trials but contributed to approximately 20% of the ESME-AMLC and CRISP cohorts) [7,8].

The role of nivolumab in the therapeutic strategy for aNSCLC evolved during the study period, with the approval of other ICIs in 2L (e.g., pembrolizumab in PD-L1 expressors > 1% in July 2016 in Germany, and in May 2017 in France) and pembrolizumab monotherapy as 1L therapy (approved in patients with high PD-L1 expression [≥50%] in January and May 2017 in Germany and France, respectively). As a result, the population of patients receiving nivolumab in clinical practice has changed. The approval of pembrolizumab with chemotherapy in September 2018 (NSQ)/March 2019 (SQ) also likely impacted the population treated with nivolumab captured in CRISP for the years 2019 and 2020. Within the study period, the proportion of PD-L1 negative patients (i.e., PD-L1 < 1%) receiving nivolumab increased over time. At the beginning of the study period (2015–2016), PD-L1 testing was minimal in patients with aNSCLC, whereas, by 2019, approximately 80% of patients had at least one PD-L1 test performed prior to starting nivolumab, and 43.1% of the patients in ESME-AMLC and 58.4% in CRISP were PD-L1-negative.

Despite the broader inclusion criteria and different periods studied, OS and PFS observed in patients receiving nivolumab in ESME-AMLC were similar with those previously reported in CheckMate 017 and CheckMate 057, as well as two French observational studies based on the national hospital database and the EVIDENS cohort (Lung Cancer Patients TrEated With NiVolumab: A LongItuDinal, ProspEctive, ObservatioNal, Multicentric Study), and the recent non-interventional prospective ENLARGE-Lung study [7,8,14,15,16]. However, in the CRISP database, while PFS were similar to those reported in previous RCTs and real-world studies, the OS observed were lower than expected [7,8,14,15,16]. The reason for this discrepancy is unclear but may reflect differences in median age between ESME-AMLC and CRISP, or potentially the change in nivolumab’s place in the therapeutic strategy, due to the availability of other ICIs in 2L from June 2016 and the approval of pembrolizumab in 1L in January 2017, as most patients (60%) receiving nivolumab in CRISP started after 2018.

In the multivariate analyses, a poorer performance status (PS) on the ECOG scale was negatively correlated with both treatment duration and OS in both cohorts, indicating poorer OS and shorter treatment duration in patients with an ECOG PS ≥ 2 compared with patients with an ECOG PS of 0 or 1. This finding aligns with results from the RCT CheckMate 153 as well as other real-world studies of nivolumab [16,17,18,19,20,21,22,23,24,25]. However, these survival rates were considered acceptable as poor PS is frequently associated with a poor prognosis regardless of treatment [26,27]. Thus, PD-L1 inhibitors, which have a more favorable safety profile than chemotherapy, may still be potential treatment options for patients with an ECOG PS ≥ 2 [23].

Time from the start of previous LoT until starting nivolumab was found to have a strong positive association with both treatment duration of nivolumab and OS in both cohorts. This is in line with the results of previously published studies showing an association between previous treatment outcomes and nivolumab efficacy [22,28,29,30]. In ESME-AMLC, the presence of *EGFR*/*ALK*/*ROS* mutations were significantly negatively associated with nivolumab treatment duration, and there was a trend toward a positive association between current and former smokers and OS when compared with never smokers. These variables were not included in CRISP analysis due to the limited sample size; however, similar findings were reported in previous trials and observational studies, suggesting that PD-L/PD-L1 inhibitors may be less active in never smokers and patients with *EGFR*/*ALK* mutations due to low mutational heterogeneity and immunogenicity. In accordance with previous studies, no negative association between patient age and OS was demonstrated, implying similar benefits from nivolumab in both elderly and younger patients [17,18,19,20,24,31]. Histology and number of prior therapy lines were also not found to impact OS and treatment duration in patients treated with nivolumab. Similar results have been reported in recent publications regarding nivolumab experience [17,24]. The absence of any distant metastasis at the time of nivolumab start was positively associated with treatment duration and OS, when compared with the presence of brain metastases in ESME-AMLC, while a similar trend was observed in CRISP for OS only, potentially due to the limited number of patients without any metastases in CRISP (n = 15). This finding aligns with poor prognosis for patients with aNSCLC observed in some studies [17,20], although this association has not been observed in others [16,18,19,24,31].

Finally, with regards to safety, nivolumab discontinuation due to toxicity represented 8% of nivolumab treated patients in ESME-AMLC and 5% in CRISP. However, this proportion might be slightly underestimated in the CRISP database, where only one reason for each treatment discontinuation could be reported (versus multiple responses accepted in ESME-AMLC), leading to a high proportion of patients having ‘other’ as the reason for nivolumab discontinuation in CRISP.

The simultaneous analysis of two substantial and methodologically similar real-world European clinical datasets on aNSCLC is a strength of this study. ESME-AMLC is a large-scale academic initiative centralizing data from multiple French hospitals involved in lung cancer management, with all patients diagnosed with aNSCLC in participating centers included in the cohort. CRISP is an academic initiative capturing data prospectively from many centers in a wide range of healthcare sectors across Germany, from the start of treatment for patients with NSCLC receiving systemic therapy in Germany. Structured electronic case reporting by trained clinical research associates and stringent quality control measures are employed in both databases to prioritize data completeness and limit inconsistencies.

The limitations of this study are generally a result of the real-world nature of the research. For instance, metastases were assessed descriptively and locally by a radiologist and not using the RECIST criteria. PFS was estimated using a proxy measure consisting of the time from the treatment line start date until the date of first disease progression identified in clinic, or the date of death for any cause, whichever occurred first. As noted above, deaths were also not cross-checked with national registries of death and, in ESME-AMLC, linkage with the national death database is planned to improve the accuracy of OS estimates in the future. Best treatment response was only available in CRISP, and was unknown in 37.0% of patients in that cohort. For this reason, the results of this study should be interpreted with caution.

Due to differences in the design of the ESME-AMLC and CRISP databases (for example, the retrospective and prospective data collection, respectively) it was not possible to aggregate populations or directly compare outcomes, and as a result this study provides commentary on trends observed in each platform only. Additionally, due to the nature of these real-world databases and an 18-month delay on data availability, the follow-up for this study was limited. In line with the improving long-term survival prospects for patients receiving ICIs, future research evaluating >3-year survival would be of interest, as would the use of ICIs in the 1L setting as they become increasingly available to patients with NSCLC. Future research could also consider investigation into predictors of adverse events, which was not within the scope of the current study.

## 5. Conclusions

The clinical outcomes of patients with aNSCLC treated with nivolumab in the real-world setting in the ESME-AMLC and CRISP databases were, overall, consistent between countries and with those previously reported in RCTs. Poor PS and a shorter time from start of previous LoT to starting nivolumab were significantly associated with shorter OS in patients treated with nivolumab in both cohorts. Future studies on real-world data sources are needed to assess patients receiving nivolumab as 2L+ with longer-term survival (>3 years) as well as those treated with immunotherapy as 1L treatment.

## Figures and Tables

**Table 1:** Demographics and clinical characteristics of patients with NSCLC treated with nivolumab in 2L+, overall and by histology in the ESME-AMLC and CRISP cohorts.

		ESME-AMLC			CRISP		
		Overall	SQ	NSQ/ Others	Overall	SQ	NSQ/ Others
		n (%)	n (%)	n (%)	n (%)	n (%)	n (%)
Cohort Size	N	2262	567	1695	522	133	389
Year of nivolumab start	2015	186 (8.2)	63 (11.1)	123 (7.3)	-	-	-
	2016	695 (30.7)	184 (32.5)	511 (30.1)	42 (8.0)	14 (10.5)	28 (7.2)
	2017	856 (37.8)	213 (37.6)	643 (37.9)	167 (32.0)	40 (30.1)	127 (32.6)
	2018	460 (20.3)	95 (16.8)	365 (21.5)	214 (41.0)	48 (36.1)	166 (42.7)
	2019/2020 *	65 (2.9)	12 (2.1)	53 (3.1)	99 (19.0)	31 (23.3)	68 (17.5)
Age (years) at nivolumab start	Median	64	66	63	66	69	64
	Q1–Q3	56.9–69.8	59.9–72.8	55.8–68.8	60.0–73.0	64.0–76.0	59.0–72.0
	≥75	262 (11.6)	106 (18.7)	156 (9.2)	126 (24.1)	46 (34.6)	80 (20.6)
Sex	Male	1560 (69.0)	465 (82.0)	1095 (64.6)	327 (62.6)	94 (70.7)	233 (59.9)
Stage at initial diagnosis	I–II	176 (7.8)	64 (11.3)	112 (6.6)	18 (3.4)	5 (3.8)	13 (3.3)
	IIIA	181 (8.0)	60 (10.6)	121 (7.1)	9 (1.7)	4 (3.0)	5 (1.3)
	IIIB–IIIC	326 (14.4)	132 (23.3)	194 (11.4)	44 (8.4)	18 (13.5)	26 (6.7)
	IV	1550 (68.5)	305 (53.8)	1245 (73.5)	428 (82)	100 (75.2)	328 (84.3)
ECOG performance status at nivolumab start	0–1	1014 (44.8)	260 (45.9)	754 (44.5)	293 (56.1)	78 (58.6)	215 (55.3)
	2	247 (10.9)	61 (10.8)	186 (11.0)	92 (17.6)	23 (17.3)	69 (17.7)
	3–4	81 (3.6)	15 (2.6)	66 (3.9)	10 (1.9)	2 (1.5)	8 (2.1)
	Missing	920 (40.7)	231 (40.7)	689 (40.6)	127 (24.3)	30 (22.6)	97 (24.9)
Smoking status at diagnosis ^†^	Never smoked	138 (6.1)	11 (1.9)	127 (7.5)	46 (8.8)	7 (5.3)	39 (10.0)
	Light former smoker	93 (4.1)	20 (3.5)	73 (4.3)	44 (8.4)	11 (8.3)	33 (8.5)
	Heavy former smoker	1139 (50.4)	317 (55.9)	822 (48.5)	200 (38.3)	56 (42.1)	144 (37.0)
	Former smoker of unknown intensity	27 (1.2)	5 (0.9)	22 (1.3)	17 (3.3)	8 (6.0)	9 (2.3)
	Smoker	804 (35.5)	200 (35.3)	604 (35.6)	160 (30.7)	38 (28.6)	122 (31.4)
	Missing	61 (2.7)	14 (2.5)	47 (2.8)	55 (10.5)	13 (9.8)	42 (10.8)
At least one metastasis at time of nivolumab start	Any location	2052 (90.7)	444 (78.3)	1608 (94.9)	507 (97.1)	129 (97.0)	378 (97.2)
	Bone	992 (43.9)	170 (30.0)	822 (48.5)	237 (45.4)	50 (37.6)	187 (48.1)
	Brain	785 (34.7)	111 (19.6)	674 (39.8)	128 (24.5)	16 (12.0)	112 (28.8)
	Symptomatic	192 (8.5)	26 (4.6)	166 (9.8)	NR	NR	NR
	Asymptomatic	593 (26.2)	85 (15.0)	508 (30.0)	NR	NR	NR
	Liver	515 (22.8)	117 (20.6)	398 (23.5)	128 (24.5)	29 (21.8)	99 (25.4)
PD-L1 testing at nivolumab start ^‡^	Testing done	678 (30.0)	114 (20.1)	564 (33.3)	310 (59.4)	76 (57.1)	234 (60.2)
	Positive	276 (12.2)	49 (8.6)	277 (16.3)	136 (26.1)	29 (21.8)	107 (27.5)
	≥50%	115 (5.1)	15 (2.6)	100 (5.9)	29 (5.6)	7 (5.3)	22 (5.7)
	1–49%	154 (6.8)	25 (4.4)	129 (7.6)	92 (17.6)	20 (15.0)	72 (18.5)
	Unknown	57 (2.5)	9 (1.6)	48 (2.8)	15 (2.9)	2 (1.5)	13 (3.3)
	Negative	352 (15.6)	65 (11.5)	287 (16.9)	170 (32.6)	47 (35.3)	123 (31.6)
	Not contributive	-	-	-	4 (0.8)	0 (0.0)	4 (1.0)
	Testing not done	1584 (70.0)	453 (79.9)	1131 (66.7)	212 (40.6)	57 (42.9)	155 (39.8)
*EGFR*	Positive	79 (3.5)	2 (0.4)	77 (4.5)	24 (4.6)	2 (1.5)	22 (5.7)
*ALK*	Positive	19 (0.8)	3 (0.5)	16 (0.9)	10 (1.9)	1 (0.8)	9 (2.3)
*ROS*	Positive	14 (0.6)	1 (0.2)	13 (0.8)	8 (1.5)	1 (0.8)	7 (1.8)
Time from initial diagnosis (months)	Median	9.6	8.8	9.9	7.8	7.7	7.9
	Q1–Q3	6.2–15.9	5.9–15.1	6.3–16.2	5.2–11.8	5.3–11.1	5.2–12.2
Line of nivolumab treatment	2L	1516 (67.0)	429 (75.7)	1087 (64.1)	433 (83.0)	122 (91.7)	311 (79.9)
	3L	554 (24.5)	121 (21.3)	433 (25.5)	75 (14.4)	8 (6.0)	67 (17.2)
	4L	141 (6.2)	16 (2.8)	125 (7.4)	11 (2.1)	3 (2.3)	8 (2.1)
	5L+	51 (2.3)	1 (0.2)	50 (2.9)	3 (0.6)	0 (0.0)	3 (0.8)
Previous regimens received (all)	PT-based CT	2052 (90.7)	507 (89.4)	1545 (91.2)	492 (94.3)	119 (89.5)	373 (95.9)
	Non-PT-based CT	506 (22.4)	105 (18.5)	401 (23.7)	43 (8.2)	9 (6.8)	34 (8.7)
	Targeted therapy	120 (5.3)	10 (1.8)	110 (6.5)	14 (2.7)	0 (0.0)	14 (3.6)
	Immunotherapy	5 (0.2)	0 (0.0)	5 (0.3)	13 (2.5)	6 (4.5)	7 (1.8)
	Investigational agents	171 (7.6)	34 (6.0)	137 (8.1)	5 (1.0)	1 (0.8)	4 (1.0)
	Other combinations	20 (0.9)	0 (0.0)	20 (1.2)	30 (5.7)	6 (4.5)	24 (6.2)
Last LoT regimen received prior to nivolumab	PT-based CT	1590 (70.3)	430 (75.8)	1160 (68.4)	439 (84.1)	115 (86.5)	324 (83.3)
	Non-PT-based CT	461 (20.4)	98 (17.3)	363 (21.4)	38 (7.3)	8 (6.0)	30 (7.7)
	Targeted therapy	66 (2.9)	10 (1.8)	56 (3.3)	4 (0.8)	0 (0.0)	4 (1.0)
	Immunotherapy	2 (0.1)	0 (0.0)	2 (0.1)	7 (1.3)	4 (3.0)	3 (0.8)
	Investigational agents	130 (5.7)	29 (5.1)	101 (6.0)	5 (1.0)	1 (0.8)	4 (1.0)
	Other combinations	13 (0.6)	0 (0.0)	13 (0.8)	29 (5.6)	5 (3.8)	24 (6.2)
Time from start of previous LoT until start of nivolumab (months)	Median	5.5	5.5	5.5	5.4	5.4	5.6
	Q1–Q3	2.9–8.6	3.0–8.0	2.9–8.9	3.2–8.1	3.4–7.8	3.0–8.2
	<3	606 (26.8)	146 (25.7)	460 (27.1)	121 (23.2)	26 (19.5)	95 (24.4)
	≥3–< 6	649 (28.7)	173 (30.5)	476 (28.1)	173 (33.1)	53 (39.8)	120 (30.8)
	≥6–<12	730 (32.3)	198 (34.9)	532 (31.4)	183 (35.1)	47 (35.3)	136 (35.0)
	≥12	277 (12.2)	50 (8.8)	227 (13.4)	44 (8.4)	7 (5.3)	37 (9.5)

* Six patients were included in 2020 for the CRISP database only. ^†^ Heavy former smoker = defined as patients who quit smoking less than 15 years ago or who quit smoking but had smoked more than 10 pack years; Light former smoker = defined as patients who quit smoking more than 15 years before diagnosis or who quit smoking and had smoked less than 10 pack years. ^‡^ Percentage based on the total number of patients with a positive PD-L1 test. In ESME-AMLC, this includes those with a positive PD-L1 test and those not contributive, whereas in CRISP, only those with a positive PD-L1 test are included. 2L, second-line; 2L+, second-line or later; 3L, third-line; 4L, fourth-line; 5L+, fifth line or later; *ALK*, anaplastic lymphoma kinase; CT, chemotherapy; ECOG, Eastern Cooperative Oncology Group; *EGFR*, epidermal growth factor receptor; LoT, line of therapy; NR, not reported; NSCLC, non-small cell lung cancer; NSQ, non-squamous; PD-L1, programmed death-ligand 1; PT, platinum; *ROS*, c-ros oncogene; SQ, squamous.

**Table 2:** Best treatment response to nivolumab in patients treated with nivolumab in 2L+ or beyond in CRISP overall, by histology and by PD-L1 status.

		Histology			PD-L1 Status at Nivolumab Start		
		Overall	SQ	NSQ/Others	Not Tested	Positive	Negative
Cohort Size	N	522	133	389	212	136	170
Best treatment response	Complete/partial response	58 (11.1%)	12 (9.0%)	46 (11.8%)	23 (10.8%)	25 (18.4%)	10 (5.9%)
	Stable disease	107 (20.5%)	27 (20.3%)	80 (20.6%)	48 (22.6%)	21 (15.4%)	37 (21.8%)
	Progressive disease	164 (31.4%)	41 (30.8%)	123 (31.6%)	58 (27.4%)	52 (38.2%)	53 (31.2%)
	Unknown	193 (37.0%)	53 (39.8%)	140 (36.0%)	83 (39.2%)	38 (27.9%)	70 (41.2%)

2L+, second-line or later; NSQ, non-squamous; PD-L1, programmed death-ligand 1; SQ, squamous.

**Table 3:** Reasons for discontinuation of nivolumab in patients treated in 2L+ in ESME-AMLC and CRISP, overall and by histology.

	ESME-AMLC			CRISP		
	Overall	SQ	NSQ/Others	Overall	SQ	NSQ/Others
Reason for Discontinuation *	1861	483	1378	522	133	389
Progression	1073 (57.7)	285 (59.0)	788 (57.2)	271 (51.9)	66 (49.6)	205 (52.7)
Toxicity	152 (8.2)	42 (8.7)	110 (8.0)	28 (5.4)	7 (5.3)	21 (5.4)
Gastrointestinal	19 (1.0)	4 (0.8)	15 (1.1)	NR	NR	NR
Hepatic	16 (0.9)	3 (0.6)	13 (0.9)	NR	NR	NR
Cutaneous	8 (0.4)	5 (1.0)	3 (0.2)	NR	NR	NR
Hematological	10 (0.5)	4 (0.8)	6 (0.4)	NR	NR	NR
Autoimmune	12 (0.6)	3 (0.6)	9 (0.7)	NR	NR	NR
Other toxicities	74 (4.0)	17 (3.5)	57 (4.1)	NR	NR	NR
Doctor’s choice/protocol driven	263 (14.1)	66 (13.7)	197 (14.3)	14 (2.7)	5 (3.8)	9 (2.3)
Patient’s death	296 (15.9)	78 (16.1)	218 (15.8)	NR	NR	NR
Patient’s choice	22 (1.2)	1 (0.2)	21 (1.5)	NR	NR	NR
Others	138 (7.4)	28 (5.8)	110 (8.0)	164 (31.4)	48 (36.1)	116 (29.8)
Missing	8 (0.4)	2 (0.4)	6 (0.4)	45 (8.6)	7 (5.3)	38 (9.8)

* Multiple answers possible in ESME-AMLC, not in CRISP. 2L+, second-line or later; NR, not reported; NSQ, non-squamous; SQ, squamous.

**Table 4:** Factors associated with treatment duration in patients treated with nivolumab in ESME-AMLC and CRISP (multivariate Cox model; table and forest plot).

		ESME-AMLC					CRISP				
Variable *		Cohort Size	Median	95% CI	HR (95% CI)	*p*-Value	Cohort Size	Median	95% CI	HR (95% CI)	*p*-Value
Age (years)	<70	1709	2.6	2.3–2.8	Reference	0.67	328	2.2	1.8–2.5	Reference	0.67
	70–74	291	2.9	2.4–3.4	1.06 (0.92–1.21)		68	2.8	2.1–3.7	0.89 (0.67–1.17)	
	≥75	262	2.4	2.1–3.1	0.98 (0.84–1.14)		126	2.6	2.0–3.2	1.01 (0.81–1.27)	
Sex	Female	702	2.5	2.3–2.8	Reference	0.55	195	2.3	1.8–2.8	Reference	0.29
	Male	1560	2.7	2.4–2.9	1.03 (0.93–1.14)		327	2.3	1.9–2.8	0.90 (0.74–1.09)	
ECOG PS	0	228	3.7	2.8–4.7	Reference	<0.01	68	2.8	2.1–3.4	Reference	0.03
	1	786	2.6	2.3–2.9	1.18 (1.00–1.39)		225	2.3	2.0–2.8	1.02 (0.76–1.36)	
	2+	328	1.4	1.3–1.8	1.87 (1.55–2.25)		102	1.4	1.0–2.3	1.29 (0.93–1.79)	
	Missing	920	3.0	2.8–3.3	1.12 (0.95–1.31)		127	2.5	1.7–3.6	0.82 (0.59–1.13)	
Smoking status	Non-smokers	138	2.0	1.6–2.4	Reference	0.16	NA				
	Former smokers	1259	2.6	2.3–2.8	0.98 (0.80–1.20)						
	Smokers	804	2.8	2.5–3.2	0.88 (0.71–1.09)						
	Missing	61	2.6	1.9–4.0	0.84 (0.60–1.19)						
Metastases	Brain	785	2.5	2.3–2.9	Reference	0.03	128	2.1	1.4–2.6	NP	NP
	Other only	1267	2.5	2.3–2.8	1.07 (0.96–1.18)		379	2.3	2.0–2.8	NP	
	No metastases	210	3.5	3.0–4.7	0.87 (0.73–1.03)		15	3.3	1.4–7.0	NP	
Time from start of previous LoT until start of nivolumab (months)	<3	606	1.9	1.8–2.3	Reference	<0.01	121	1.4	1.1–2.1	Reference	<0.01
	3–6	649	2.3	1.9–2.4	0.99 (0.87–1.11)		173	2.1	1.4–2.4	0.78 (0.61–1.01)	
	6–12	730	3.1	2.8–3.5	0.80 (0.71–0.91)		183	2.8	2.3–3.5	0.65 (0.50–0.84)	
	≥12	277	4.4	3.5–5.5	0.63 (0.53–0.75)		44	4.6	3.2–7.9	0.55 (0.37–0.82)	
PD-L1 expression	Positive	276	2.8	2.3–3.4	Reference	0.03	136	1.9	1.5–2.8	Reference	0.06
	Negative	352	2.3	1.8–2.7	1.24 (1.03–1.48)		170	2.3	1.7–2.8	1.31 (1.01–1.68)	
	Unknown	1634	2.8	2.5–2.9	1.04 (0.90–1.20)		216	2.4	2.2–3.2	1.29 (1.02–1.64)	
*EGFR*, *ALK* or *ROS* mutations	No	2154	2.7	2.5–2.9	Reference	<0.01	NA				
	Yes	108	2.0	1.5–2.3	1.50 (1.20–1.87)						
Year of nivolumab start	2015	186	2.2	1.9–2.8	Reference	0.17					
	2016	695	2.6	2.3–3.0	0.99 (0.83–1.17)		42	2.3	1.0–5.2	Reference	0.08
	2017	856	2.5	2.3–2.8	1.00 (0.84–1.18)		167	2.6	1.9–3.5	1.11 (0.77–1.59)	
	2018	460	3.1	2.4–3.5	0.91 (0.75–1.11)		214	1.9	1.4–2.3	1.42 (1.00–2.04)	
	2019/2020	65	6.2	2.3–NE	0.60 (0.38–0.94)		99	3.1	2.4–3.7	1.30 (0.86–1.98)	
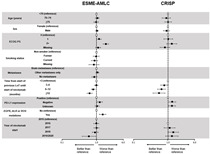											

* The model was adjusted for the year of nivolumab start and line of nivolumab treatment for ESME-AMLC, and BMI and line of nivolumab treatment for CRISP. Forest plot data are hazard ratios (with 95% confidence intervals) compared with the reference for each variable. *ALK*, anaplastic lymphoma kinase; CI, confidence interval; ECOG PS, Eastern Cooperative Oncology Group performance score; EGFR, epidermal growth factor receptor; HR, hazard ratio; LoT, line of therapy; NA, not available; NP, not provided (i.e., *p*-value < 0.2 in the univariate analysis); PD-L1, programmed cell death ligand 1.

**Table 5:** Factors associated with overall survival in patients treated with nivolumab in ESME-AMLC and CRISP (multivariate Cox model; table and forest plot).

		ESME-AMLC					CRISP				
Variable *		Cohort Size	Median	95% CI	HR (95% CI)	*p*-Value	Cohort Size	Median	95% CI	HR (95% CI)	*p*-Value
Age (years)	<70	1709	12.2	10.7–13.9	Reference	0.39	328	7.3	5.4–8.5	Reference	0.08
	70–74	291	12.6	9.2–14.8	1.08 (0.90–1.29)		68	8.0	5.3–18.9	0.71 (0.51–1.01)	
	≥75	262	9.7	7.5–12.6	1.12 (0.93–1.34)		126	7.8	6.1–11.4	1.11 (0.86–1.44)	
Sex	Female	702	13.9	11.0–16.0	Reference	0.04	195	8.0	5.8–9.9	Reference	0.58
	Male	1560	11.2	10.0–12.8	1.15 (1.01–1.31)		327	7.4	5.8–8.9	0.94 (0.75–1.17)	
ECOG PS	0	228	19.8	17.0–27.1	Reference	<0.01	68	11.8	7.9–15.3	Reference	<0.01
	1	786	10.7	9.4–12.5	1.73 (1.38–2.15)		225	7.8	6.2–9.3	1.22 (0.86–1.73)	
	2+	328	3.7	2.9–4.3	3.24 (2.55–4.12)		102	3.9	2.5–5.5	2.00 (1.36–2.94)	
	Missing	920	15.4	13.2–18.0	1.42 (1.13–1.77)		127	7.6	5.3–13.2	1.07 (0.73–1.57)	
Smoking status	Non-smoker	138	10.7	8.0–17.3	Reference	0.07	46	6.8	3.9–17.0	NP	NA
	Former	1259	10.7	9.9–12.4	0.96 (0.76–1.22)		261	8.1	5.8–11.3	NP	
	Current	804	14.7	11.6–16.6	0.82 (0.64–1.06)		160	7.2	5.0–8.1	NP	
	Missing	61	14.6	7.6–23.8	0.75 (0.48–1.15)		55	8.9	5.2–12.7	NP	
Metastases	Brain	785	13.0	11.0–15.1	Reference	0.05	128	7.1	4.1–8.5	Reference	0.17
	Other	1267	10.5	9.2–12.5	1.07 (0.94–1.22)		379	7.6	6.2–9.6	0.90 (0.70–1.17)	
	No metastases	210	14.5	12.0–17.3	0.84 (0.67–1.04)		15	23.7	5.3–NA	0.44 (0.19–1.05)	
Time from start of previous lot until start of nivolumab (months)	<3	606	7.0	6.0–8.3	Reference	<0.01	121	4.0	2.8–6.1	Reference	<0.01
	3–6	649	9.8	8.4–11.6	0.86 (0.75–1.00)		173	6.7	5.0–7.9	0.75 (0.57–1.00)	
	6–12	730	15.4	14.0–18.5	0.61 (0.53–0.71)		183	10.9	7.7–13.4	0.57 (0.43–0.75)	
	≥12	277	23.8	18.0–28.0	0.44 (0.35–0.55)		44	12.8	6.2–19.7	0.48 (0.30–0.77)	
PD-L1 expression	Positive	276	13.0	9.4–18.4	NP	NA	136	10.8	5.8–13.3	Reference	<0.01
	Negative	352	10.8	8.4–15.8	NP		170	6.1	4.6–7.7	1.66 (1.24–2.23)	
	Unknown	1634	12.0	10.6–13.3	NP		216	7.8	6.1–8.9	1.39 (1.06–1.82)	
Stage at diagnosis	I–II	176	10.9	8.7–15.2	NP	NA	18	27.3	6.9–NA	Reference	0.14
	III	507	13.4	11.2–16.0	NP		53	12.1	6.2–18.0	2.02 (0.88–4.62)	
	IV	1550	11.1	10.0–13.0	NP		428	7.2	5.5–8.0	2.38 (1.12–5.07)	
	Missing	0	NA	NA	NA		23	7.2	4.8–11.4	2.19 (0.88–5.48)	
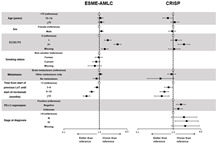											

* The model was adjusted for line of nivolumab treatment (for ESME-AMLC). Forest plot data are hazard ratios (with 95% confidence intervals) compared with the reference for each variable. CI, confidence interval; ECOG PS, Eastern Cooperative Oncology Group performance score; HR, hazard ratio; LoT, line of therapy; NA, not available; NP, not provided (i.e., *p*-value < 0.2 in the univariate analysis); PD-L1, programmed cell death ligand 1.

## Data Availability

The data presented in this study are available on reasonable request.

## Supplementary Materials

The following supporting information can be downloaded at: https://www.mdpi.com/article/10.3390/cancers14246148/s1. Table S1: Demographics and clinical characteristics of patients with NSCLC treated with nivolumab in 2L+ in ESME-AMLC and CRISP cohort over time (i.e., by year of nivolumab start). Table S2: Factors associated with overall survival in patients treated with nivolumab in ESME-AMLC. Table S3: Factors associated with overall survival in patients treated with nivolumab in CRISP. Table S4: Factors associated with treatment duration in patients treated with nivolumab in ESME-AMLC. Table S5: Factors associated with treatment duration in patients treated with nivolumab in CRISP. Figure S1: Sensitivity analysis for OS by histology in ESME-AMLC.
