# Supplementary material for: Effectiveness of Nivolumab in Second-Line and Later in Patients with Advanced Non-Small Cell Lung Cancer in Real-Life Practice in France and Germany: Analysis of the ESME-AMLC and CRISP Cohorts
Source: Cancers (Basel). 2022 Dec 13;14(24):6148. doi: 10.3390/cancers14246148 (PMC9776880; doi:10.3390/cancers14246148)
Supplement: Supplementary file 1 [file cancers-14-06148-s001.zip › cancers-1985706-supplementary.pdf]

Supplementary Material

Table S1: Demographics and clinical characteristics of patients with NSCLC treated with nivolumab in 2L+ in ESME-AMLC and CRISP cohort over time (i.e., by year of nivolumab start)

		ESME-AMLC					CRISP			
		**2015**	**2016**	**2017**	**2018**	**2019**	**2016**	**2017**	**2018**	**2019**
**Cohort size**	**N**	**186**	**695**	**856**	**460**	**65**	**42**	**167**	**214**	**93**
**Age (years)**	**Median**	61.3	63.5	63.4	64.2	67.1	64.5	65.0	65.0	67.0
	**Q1–Q3**	54.9–67.4	56.7–69.3	57.0–69.8	58.0–71.3	59.0–72.0	61.0–74.0	59.0–74.0	59.0–72.0	62.0–74.0
	**Range**	27.8–83.9	33.3–91.8	32.1–89.5	36.1–89.3	44.7–85.4	42.0–82.0	41.0–86.0	45.0–86.0	46.0–88.0
**Sex**	**Male**	134 (72.0)	503 (72.4)	571 (66.7)	304 (66.1)	48 (73.8)	23 (54.8)	107 (64.1)	135 (63.1)	60 (64.5)
**ECOG PS**	**0–1**	84 (45.2)	313 (45.0)	372 (43.4)	211 (45.9)	34 (52.3)	22 (52.4)	91 (54.5)	122 (57.0)	56 (60.2)
	**2**	20 (10.7)	61 (8.8)	106 (12.4)	52 (11.3)	8 (17.4)	9 (21.4)	37 (22.2)	33 (15.4)	12 (12.9)
	**3–4**	8 (4.3)	19 (2.7)	33 (3.8)	17 (3.7)	<5	<5	<5	<5	0
	**Missing**	74 (39.8)	302 (43.4)	345 (40.3)	180 (39.1)	19 (29.2)	9 (21.4)	35 (21.0)	55 (25.7)	25 (26.9)
**Histology**	**Adenocarcinoma**	99 (53.2)	432 (62.2)	549 (64.1)	324 (70.4)	50 (76.9)	25 (59.5)	114 (68.3)	155 (72.4)	58 (62.4)
	**Large cell carcinoma**	<5	19 (2.7)	13 (1.5)	11 (2.4)	<5	<5	5 (3.0)	<5	<5
	**Undifferentiated carcinoma**	7 (3.8)	15 (2.2)	22 (2.6)	5 (1.1)	0	NR	NR	NR	NR
	**Squamous**	63 (33.9)	184 (26.5)	213 (24.9)	95 (20.7)	12 (18.5)	14 (33.3)	40 (24.0)	48 (22.4)	30 (32.3)
	**Others**	14 (7.5)	45 (6.5)	59 (6.9)	25 (5.4)	<5	<5	8 (4.8)	10 (4.7)	<5
**At least one metastasis**		169 (90.9)	638 (91.8)	775 (90.5)	412 (89.6)	58 (89.2)	42 (100.0)	163 (97.6)	204 (95.3)	92 (98.9)
**Metastases locations**	**Bone metastases**	91 (48.9)	305 (43.9)	366 (42.8)	198 (43.0)	32 (49.2)	16 (38.1)	77 (46.1)	100 (46.7)	41 (44.1)
	**Brain metastases**						11 (26.2)	40 (24.0)	54 (25.2)	20 (21.5)
	- **Symptomatic brain met**	10 (5.4)	57 (8.2)	80 (9.3)	41 (8.9)	<5	NR	NR	NR	NR

		ESME-AMLC					CRISP			
		2015	2016	2017	2018	2019	2016	2017	2018	2019
**Cohort size**	**N**	**186**	**695**	**856**	**460**	**65**	**42**	**167**	**214**	**93**
	- **Asymptomatic brain met**	35 (18.8)	168 (24.2)	237 (27.7)	133 (28.9)	20 (30.8)	NR	NR	NR	NR
	**Liver metastases**	46 (24.7)	170 (24.5)	193 (22.5)	90 (19.6)	16 (24.6)	6 (14.3)	44 (26.3)	57 (26.6)	19 (20.4)
**PD-L1 testing done**	**Testing done**	18 (9.7)	110 (15.8)	234 (27.3)	266 (57.8)	50 (76.9)	12 (28.6)	72 (43.1)	149 (69.6)	74 (79.6)
	**Positive**	12 (6.5)	48 (6.9)	114 (13.3)	82 (17.8)	20 (30.8)	8 (19.0)	34 (20.4)	70 (32.7)	23 (24.7)
	**1–49%**	6 (3.2)	25 (3.6)	54 (6.3)	57 (12.4)	12 (18.5)	<5	19 (11.4)	55 (25.7)	16 (17.2)
	**≥50%**	<5	27 (3.9)	55 (6.4)	24 (5.2)	6 (9.2)	<5	12 (7.2)	11 (5.1)	<5
	**Unknown expression level**	<5	10 (1.4)	25 (2.9)	15 (15.6)	4 (18.2)	<5	<5	<5	<5
	**Negative**	6 (3.2)	48 (6.9)	100 (11.7)	170 (37.0)	28 (43.1)	<5	38 (22.8)	76 (35.5)	51 (58.4)
	**Not done**	168 (90.3)	585 (84.2)	622 (72.7)	194 (42.2)	15 (23.1)	30 (71.4)	95 (56.9)	65 (30.4)	19 (20.4)
EGFR	**Positive**	<5	16 (2.3)	35 (4.1)	17 (3.7)	7 (10.8)	<5	6 (3.6)	8 (3.7)	7 (7.5)
ALK	**Positive**	<5	6 (0.9)	8 (0.9)	<5	<5	<5	<5	5 (2.3)	<5
ROS	**Positive**	0 (0.0)	7 (1.0)	5 (0.6)	<5	0	0	<5	<5	<5
**Time from initial diag. to starting nivolumab (months)**	**Median**	6.3	9.3	9.8	12.0	12.2	6.1	7.1	7.2	11.3
	**Q1–Q3**	4.8–8.4	6.4–13.3	6.0–16.7	7.5–21.5	8.3–22.3	3.8–9.0	4.8–10.5	5.0–11.3	7.6–15.9
**Line of nivolumab treatment**	**2L**	140 (75.3)	460 (66.2)	568 (66.4)	308 (67.0)	40 (61.5)	35 (83.3)	145 (86.8)	178 (83.2)	73 (78.5)
	**3L**	42 (22.6)	167 (24.0)	218 (25.5)	111 (24.1)	16 (24.6)	7 (16.7)	22 (13.2)	28 (13.1)	16 (17.2)
	**4L**	<5	49 (7.1)	54 (6.3)	28 (6.1)	7 (10.8)	0	0	5 (2.3)	<5
	**5L+**	<5	19 (2.7)	16 (1.9)	13 (2.8)	<5	0	0	<5	0
**Previous regimens**	**Platinum (PT)-based chemotherapy (CT)**	159 (85.5)	616 (88.6)	784 (91.6)	430 (93.5)	63 (96.9)	37 (88.1)	161 (96.4)	205 (95.8)	84 (90.3)

		ESME-AMLC					CRISP			
		**2015**	**2016**	**2017**	**2018**	**2019**	**2016**	**2017**	**2018**	**2019**
**Cohort size**	**N**	**186**	**695**	**856**	**460**	**65**	**42**	**167**	**214**	**93**
**received (all)**	**Non-PT based CT**	33 (17.7)	165 (23.7)	195 (22.8)	101 (22.0)	12 (18.5)	5 (11.9)	10 (6.0)	16 (7.5)	10 (10.8)
	**EGFR/ALK targeted therapy**	7 (3.8)	33 (4.7)	51 (6.0)	19 (4.1)	10 (15.4)	<5	<5	9 (4.2)	<5
	**Immunotherapy**	0	0	<5	<5	<5	<5	<5	5 (2.3)	5 (5.4)
	**Investigational agents**	16 (8.6)	74 (10.6)	58 (6.8)	21 (4.6)	2 (3.1)	0	0	<5	<5
	**Other combinations**	<5	7 (1.0)	6 (0.7)	6 (1.3)	0	<5	10 (6.0)	12 (5.6)	6 (6.5)
**Last LoT regimen received prior to nivolumab**	**PT-based CT**	132 (71.0)	467 (67.2)	599 (70.0)	341 (74.1)	51 (78.5)	35 (83.3)	144 (86.2)	184 (86.0)	73 (78.5)
	**Non-PT based CT**	30 (16.1)	146 (21.0)	183 (21.4)	92 (20.0)	10 (15.4)	5 (11.9)	10 (6.0)	14 (6.5)	9 (9.7)
	**EGFR/ALK targeted therapy**	7 (3.8)	18 (2.6)	31 (3.6)	7 (1.5)	<5	0	<5	<5	<5
	**Immunotherapy**	0	0	<5	<5	0	<5	<5	<5	<5
	**Investigational agents**	16 (8.6)	60 (8.6)	39 (4.6)	14 (3.0)	<5	0	0	<5	<5
	**Other combinations**	<5	<5	<5	5 (1.1)	0	<5	9 (5.4)	12 (5.6)	6 (6.5)
**Time from start of previous LoT until start of nivolumab (months)**	**Median**	3.7	5.1	5.6	6.9	6.5	3.3	5.1	5.2	7.7
	**Q1–Q3**	2.3–5.7	2.8–8.0	2.9–8.9	3.7–11.0	4.5–9.2	2.1–5.8	3.0–7.7	3.1–7.4	5.2–11.8
	**<3**	70 (37.6)	204 (29.4)	232 (27.1)	87 (18.9)	13 (20.0)	18 (42.9)	43 (25.7)	51 (23.8)	8 (8.6)
	**≥3–<6**	78 (41.9)	214 (30.8)	227 (26.5)	116 (25.2)	14 (21.5)	14 (33.3)	57 (34.1)	78 (36.4)	22 (23.7)
	**≥6–<12**	38 (20.4)	229 (32.9)	274 (32.0)	163 (35.4)	26 (40.0)	10 (23.8)	56 (33.5)	74 (34.6)	42 (45.2)
	**≥12**	0	48 (6.9)	123 (14.4)	94 (20.4)	12 (18.5)	0	11 (6.6)	10 (4.7)	21 (22.6)

2L, second-line; 2L+, second-line or later; 3L, third-line; 4L, fourth-line; 5L+, fifth-line or later; *ALK*, anaplastic lymphoma kinase; CI, confidence interval; ECOG PS, Eastern Cooperative Oncology Group performance score; *EGFR*, epidermal growth factor receptor; HR, hazard ratio; LoT, line of therapy; NR, not reported; NSCLC, non-small cell lung cancer; PD-L1, programmed death-ligand 1; PT, platinum; *ROS*, c-ros oncogene.

Table S2: Factors associated with overall survival in patients treated with nivolumab in ESME-AMLC

Risk factor*	Cohort size	OS		Univariate Cox regression		Multivariate Cox regression	
		Median	95% CI	HR (95% CI)	p-value	HR (95% CI)	p-value
**Age (years)**					0.16		0.39
<70	1,709	12.2	10.7–13.9				
70−74	291	12.6	9.2–14.8	1.05 (0.88–1.25)		1.08 (0.90–1.29)	
≥75	262	9.7	7.5–12.6	1.18 (0.99–1.41)		1.12 (0.93–1.34)	
**Sex**					0.04		0.04
Female	702	13.9	11.0–16.0				
Male	1,560	11.2	10.0–12.8	1.14 (1.01–1.29)		1.15 (1.01–1.31)	
**ECOG PS**					<0.01		<0.01
0	228	19.8	17.0–27.1				
1	786	10.7	9.4–12.5	1.78 (1.43–2.22)		1.73 (1.38–2.15)	
2+	328	3.7	2.9–4.3	3.48 (2.74–4.42)		3.24 (2.55–4.12)	
Missing	920	15.4	13.2–18.0	1.41 (1.13–1.76)		1.42 (1.13–1.77)	
**Smoking status**					0.06		0.07
Non-smokers	138	10.7	8.0–17.3				
Former smokers	1,259	10.7	9.9–12.4	0.95 (0.75–1.20)		0.96 (0.76–1.22)	
Current smokers	804	14.7	11.6–16.6	0.82 (0.64–1.04)		0.82 (0.64–1.06)	
Missing	61	14.6	7.6–23.8	0.77 (0.50–1.19)		0.75 (0.48–1.15)	
**Number of comorbidities at inclusion**					0.26		
0	1,283	10.7	9.6–13.0				
1	691	13.2	11.6–15.4	0.90 (0.79–1.02)			
2+	288	12.1	9.2–14.8	0.99 (0.83–1.18)			
**Stage at diagnosis**					0.53		
I−II	176	10.9	8.7–15.2				
III	507	13.4	11.2–16.0	0.95 (0.74–1.22)			
IV	1,550	11.1	10.0–13.0	1.04 (0.83–1.31)			
Missing	29	16.6	3.2– NE	0.86 (0.50–1.49)			
**PD-L1 expression**					0.49		
Positive	276	13.0	9.4–18.4				
Negative	352	10.8	8.4–15.8	1.14 (0.91–1.43)			
Unknown	1,634	12.0	10.6–13.3	1.06 (0.88–1.27)			
**Year of nivolumab start**					0.68		
2015	186	9.9	7.0–14.9				
2016	856	12.1	10.4–14.3	0.93 (0.76–1.14)			
2017	460	11.6	9.7– NE	0.94 (0.74–1.19)			
2018	695	12.5	10.0–13.9	0.96 (0.79–1.18)			
2019	65	NR	NE – NE	0.60 (0.29–1.23)			
**Histology**					0.32		
Squamous	567	10.5	9.6–12.5				
All others	1,695	12.6	10.8–14.2	0.94 (0.82–1.07)			
**Metastases**					0.04		0.05
Brain metastases	785	13.0	11.0–15.1				
Other metastases only	1,267	10.5	9.2–12.5	1.12 (0.99–1.27)		1.07 (0.94–1.22)	
No metastases	210	14.5	12.0–17.3	0.89 (0.72–1.11)		0.84 (0.67–1.04)	
**Treatment line**					0.11		0.49
2	1,516	13.2	11.2–14.5				
3+	746	10.4	9.0–12.2	1.10 (0.98–1.24)		0.96 (0.84–1.09)	
**Time from start of previous LoT until start of nivolumab**					<0.01		<0.01
<3 months	606	7.0	6.0–8.3				
≥3–<6 months	649	9.8	8.4–11.6	0.85 (0.74–0.98)		0.86 (0.75–1.00)	
≥6–<12 months	730	15.4	14.0–18.5	0.60 (0.52–0.70)		0.61 (0.53–0.71)	
≥12 months	277	23.8	18.0–28.0	0.41 (0.33–0.52)		0.44 (0.35–0.55)	
**Autoimmune disease**					0.58		
No (Ref)	2,209	12.1	10.7–13.3				
Yes	53	9.2	5.5–16.2	1.11 (0.77–1.60)			
***EGFR*, *ALK* or *ROS* mutations**					0.56		
No (Ref)	2,154	12.2	10.7–13.3				
Yes	108	10.7	7.6–16.0	1.08 (0.83–1.40)			

*Characteristics at time of nivolumab start if not specified.

ALK, anaplastic lymphoma kinase; BMI, body mass index; CI, confidence interval; ECOG PS, Eastern Cooperative Oncology Group performance score; *EGFR*, epidermal growth factor receptor; HR, hazard ratio; LoT, line of therapy; OS, overall survival; PD-L1, programmed death-ligand 1; *ROS*, c-ros oncogene.

Table S3: Factors associated with overall survival in patients treated with nivolumab in CRISP

Variable*		Cohort size	OS Median	OS 95% CI	Univariate Cox regression HR (95% CI)	Univariate p-value	Multivariate Cox regression HR (95% CI)	Multivariate p-value
**Age (years)**						0.16		0.08
	<70	328	7.3	5.4–8.5	Reference		Reference	
	70–74	68	8.0	5.3–18.9	0.72 (0.51–1.01)		0.71 (0.51–1.01)	
	≥75	126	7.8	6.1–11.4	0.98 (0.76–1.25)		1.11 (0.86–1.44)	
**Sex**						0.68		0.58
	Female	195	8.0	5.8–9.9	Reference		Reference	
	Male	327	7.4	5.8–8.9	0.96 (0.77–1.18)		0.94 (0.75–1.17)	
**ECOG PS**						<0.01		<0.01
	0	68	11.8	7.9–15.3	Reference		Reference	
	1	225	7.8	6.2–9.3	1.21 (0.86–1.71)		1.22 (0.86–1.73)	
	2+	102	3.9	2.5–5.5	1.88 (1.29–2.75)		2.00 (1.36–2.94)	
	Missing	127	7.6	5.3–13.2	1.15 (0.79–1.68)		1.07 (0.73–1.57)	
**BMI**						0.72		
	Underweight	30	7.7	2.0–19.4	Reference			
	Normal weight	281	7.6	5.6–11.3	0.90 (0.57–1.42)			
	Overweight	145	6.2	5.0–8.1	1.05 (0.65–1.69)			
	Obesity	57	8.7	6.1–12.4	0.86 (0.51–1.47)			
	Missing	9	8.8	1.0–NA	0.81 (0.31–2.17)			
**Number of comorbidities at inclusion**						0.61		
	0	192	7.0	4.9–8.7	Reference			
	1	197	8.1	6.2–10.9	0.89 (0.70–1.13)			
	2+	133	7.4	5.4–12.0	0.96 (0.74–1.26)			
**Smoking status**						0.99		
	Non-smoker	46	6.8	3.9–17.0	Reference			
	Former smoker	261	8.1	5.8–11.3	0.97 (0.67–1.42)			
	Current smoker	160	7.2	5.0–8.1	1.01 (0.68–1.51)			
	Missing	55	8.9	5.2–12.7	0.99 (0.61–1.59)			
**Stage at diagnosis**						0.02		0.14
	I–II	18	27.3	6.9–NA	Reference		Reference	
	III	53	12.1	6.2–18.0	1.81 (0.80–4.07)		2.02 (0.88–4.62)	
	IV	428	7.2	5.5–8.0	2.56 (1.21–5.42)		2.38 (1.12–5.07)	
	Missing	23	7.2	4.8–11.4	2.78 (1.13–6.84)		2.19 (0.88–5.48)	
**PD-L1 expression**						0.1		<0.01
	Positive	136	10.8	5.8–13.3	Reference		Reference	
	Negative	170	6.1	4.6–7.7	1.34 (1.01–1.78)		1.66 (1.24–2.23)	
	Unknown	216	7.8	6.1–8.9	1.27 (0.98–1.66)		1.39 (1.06–1.82)	
**Year of nivolumab start**						0.27		
	2016	42	10.4	3.4–16.1	Reference			
	2017	167	8.7	6.1–12.6	0.98 (0.66–1.46)			
	2018	214	6.1	5.1–8.0	1.24 (0.84–1.83)			
	2019/2020	99	7.6	6.2–11.3	1.05 (0.67–1.64)			
**Histology**						0.61		
	Squamous	133	7.9	6.1–11.6	Reference			
	All others	389	7.4	5.8–8.7	1.06 (0.84–1.35)			
**Metastases**						0.06		0.17
	Brain metastases	128	7.1	4.1–8.5	Reference		Reference	
	Other metastases only	379	7.6	6.2–9.6	0.92 (0.72–1.18)		0.90 (0.70–1.17)	
	No metastases	15	23.7	5.3–NA	0.40 (0.18–0.86)		0.44 (0.19–1.05)	
**Treatment line**						0.64		
	2	433	7.7	6.3–8.9	Reference			
	3+	89	6.1	4.1–11.3	1.07 (0.80–1.43)			
**Time from start of previous LoT until start of nivolumab**						<0.01		<0.01
	<3 months	121	4.0	2.8–6.1	Reference		Reference	
	≥3–<6 months	173	6.7	5.0–7.9	0.79 (0.60–1.03)		0.75 (0.57–1.00)	
	≥6–<12 months	183	10.9	7.7–13.4	0.60 (0.45–0.78)		0.57 (0.43–0.75)	
	≥12 months	44	12.8	6.2–19.7	0.48 (0.30–0.75)		0.48 (0.30–0.77)	

*Characteristics at time of nivolumab start if not specified.

BMI, body mass index; CI, confidence interval; ECOG PS, Eastern Cooperative Oncology Group performance score; HR, hazard ratio; LoT, line of therapy; OS, overall survival; PD-L1, programmed death-ligand 1.

Table S4: Factors associated with treatment duration in patients treated with nivolumab in ESME-AMLC

	Cohort	Treatment duration		Univariable Cox regression		Multivariable Cox regression	
**Risk factor***	**size**	**Median**	**95% CI**	**HR (95% CI)**	**p-value**	**HR (95% CI)**	**p-value**
**Age (years)**					0.54		0.67
<70	1,709	2.6	2.3–2.8				
70–74	291	2.9	2.4–3.4	1.07 (0.94–1.23)		1.06 (0.92–1.21)	
≥75	262	2.4	2.1–3.1	1.05 (0.91–1.21)		0.98 (0.84–1.14)	
**Sex**					0.76		0.55
Female	702	2.5	2.3–2.8				
Male	1560	2.7	2.4–2.9	1.02 (0.92–1.12)		1.03 (0.93–1.14)	
**ECOG PS**					<0.01		<0.01
0	228	3.7	2.8–4.7				
1	786	2.6	2.3–2.9	1.21 (1.03–1.42)		1.18 (1.00–1.39)	
2+	328	1.4	1.3–1.8	1.94 (1.61–2.33)		1.87 (1.55–2.25)	
Missing	920	3.0	2.8–3.3	1.11 (0.95–1.30)		1.12 (0.95–1.31)	
**Smoking status**					0.06		0.16
Non-smokers	138	2.0	1.6–2.4				
Former smokers	1,259	2.6	2.3–2.8	0.86 (0.71–1.04)		0.98 (0.80–1.20)	
Smokers	804	2.8	2.5–3.2	0.78 (0.64–0.95)		0.88 (0.71–1.09)	
Missing	61	2.6	1.9–4.0	0.78 (0.55–1.10)		0.84 (0.60–1.19)	
**Number of comorbidities at inclusion**					0.67		
0	1,283	2.4	2.3–2.8				
1	691	2.9	2.5–3.2	0.96 (0.87–1.06)			
2+	288	2.6	2.3–3.2	1.01 (0.88–1.16)			
**Stage at diagnosis**					0.62		
I–II	176	2.8	2.3–3.3				
III	507	2.8	2.4–3.2	0.96 (0.79–1.16)			
IV	1,550	2.5	2.3–2.8	0.99 (0.83–1.17)			
Missing	29	2.8	1.8–6.0	0.75 (0.48–1.18)			
**PD-L1 expression**					0.04		0.03
Positive	276	2.8	2.3–3.4				
Negative	352	2.3	1.8–2.7	1.19 (1.00–1.42)		1.24 (1.03–1.48)	
Unknown	1,634	2.8	2.5–2.9	1.01 (0.87–1.16)		1.04 (0.90–1.20)	
**Year of nivolumab start**					0.10		0.17
2015	186	2.2	1.9–2.8				
2016	695	2.6	2.3–3.0	0.92 (0.78–1.09)		0.99 (0.83–1.17)	
2017	856	2.5	2.3–2.8	0.91 (0.77–1.08)		1.00 (0.84–1.18)	
2018	460	3.1	2.4–3.5	0.83 (0.69–1.00)		0.91 (0.75–1.11)	
2019	65	6.2	2.3–NE	0.60 (0.39–0.94)		0.60 (0.38–0.94)	
**Histology**					0.32		
Squamous	567	2.8	2.5–3.2				
All others	1,695	2.5	2.3–2.8	0.95 (0.86–1.05)			
**Metastases**					0.01		0.03
Brain metastases	785	2.5	2.3–2.9				
Other metastases only	1,267	2.5	2.3–2.8	1.10 (0.99–1.21)		1.07 (0.96–1.18)	
No metastases	210	3.5	3.0–4.7	0.88 (0.74–1.05)		0.87 (0.73–1.03)	
**Treatment line**					0.01		0.43
2	1,516	2.9	2.6–3.1				
3+	746	2.3	2.0–2.5	1.13 (1.03–1.25)		1.04 (0.94–1.15)	
**Time from start of previous LoT until start of nivolumab**					<0.01		<0.01
<3 months	606	1.9	1.8–2.3				
≥3–<6 months	649	2.3	1.9–2.4	0.95 (0.84–1.07)		0.99 (0.87–1.11)	
≥6–<12 months	730	3.1	2.8–3.5	0.77 (0.68–0.86)		0.80 (0.71–0.91)	
≥12 months	277	4.4	3.5–5.5	0.59 (0.50–0.69)		0.63 (0.53–0.75)	
**Autoimmune disease**					0.38		
No (Ref)	2,209	2.6	2.4–2.8				
Yes	53	2.6	1.8–3.9	1.14 (0.85–1.53)			
***EGFR*, *ALK* or *ROS* mutations**					<0.01		<0.01
No (Ref)	2154	2.7	2.5–2.9				
Yes	108	2.0	1.5–2.3	1.48 (1.20–1.82)		1.50 (1.20–1.87)	

***** Characteristics at time of nivolumab start if not specified

ALK, anaplastic lymphoma kinase; BMI, body mass index; CI, confidence interval; ECOG PS, Eastern Cooperative Oncology Group performance score; *EGFR*, epidermal growth factor receptor; HR, hazard ratio; LoT, line of therapy; PD-L1, programmed death-ligand 1; ROS, c-ros oncogene.

Table S5: Factors associated with treatment duration in patients treated with nivolumab in CRISP

Variable*	Cohort size	Treatment duration		Univariable Cox regression		Multivariable Cox regression	
		Median	95% CI	HR (95% CI)	p-value	HR (95% CI)	p-value
**Age (years)**					0.63		0.67
<70	328	2.2	1.8–2.5	Reference		Reference	
70–74	68	2.8	2.1–3.7	0.87 (0.67–1.15)		0.89 (0.67–1.17)	
≥75	126	2.6	2.0–3.2	0.97 (0.78–1.20)		1.01 (0.81–1.27)	
**Sex**					0.5		0.29
Female	195	2.3	1.8–2.8	Reference		Reference	
Male	327	2.3	1.9–2.8	0.94 (0.78–1.13)		0.90 (0.74–1.09)	
**ECOG PS**					0.13		0.03
0	68	2.8	2.1–3.4	Reference		Reference	
1	225	2.3	2.0–2.8	1.04 (0.78–1.38)		1.02 (0.76–1.36)	
2+	102	1.4	1.0–2.3	1.25 (0.91–1.72)		1.29 (0.93–1.79)	
Missing	127	2.5	1.7–3.6	0.90 (0.66–1.23)		0.82 (0.59–1.13)	
**BMI**					0.15		0.29
Underweight	30	1.5	1.0–3.7	Reference		Reference	
Normal weight	281	2.3	1.7–2.8	1.03 (0.69–1.53)		1.16 (0.77–1.76)	
Overweight	145	2.3	1.9–2.8	1.18 (0.78–1.78)		1.36 (0.88–2.10)	
Obesity	57	3.5	2.1–4.6	0.85 (0.53–1.36)		1.07 (0.65–1.74)	
Missing	9	6.0	0.6–NA	0.51 (0.19–1.31)		0.72 (0.27–1.93)	
**Number of comorbidities at inclusion**					0.93		
0	192	2.1	1.8–2.6	Reference			
1	197	2.7	2.3–3.1	0.96 (0.78–1.19)			
2+	133	2.3	1.4–2.8	0.99 (0.79–1.26)			
**Stage at diagnosis**					0.34		
I–II	18	4.6	2.3–10.2	Reference			
III	53	2.6	1.5–3.7	1.55 (0.87–2.76)			
IV	428	2.3	1.9–2.6	1.59 (0.95–2.66)			
Missing	23	1.8	1.0–3.9	1.75 (0.90–3.40)			
**PD-L1 expression**					0.14		0.06
Positive	136	1.9	1.5–2.8	Reference		Reference	
Negative	170	2.3	1.7–2.8	1.27 (1.00–1.61)		1.31 (1.01–1.68)	
Unknown	216	2.4	2.2–3.2	1.20 (0.95–1.51)		1.29 (1.02–1.64)	
**Year of nivolumab start**					0.18		0.08
2016	42	2.3	1.0–5.2	Reference		Reference	
2017	167	2.6	1.9–3.5	1.03 (0.73–1.47)		1.11 (0.77–1.59)	
2018	214	1.9	1.4–2.3	1.26 (0.89–1.77)		1.42 (1.00–2.04)	
2019/2020	99	3.1	2.4–3.7	1.02 (0.69–1.50)		1.30 (0.86–1.98)	
**Histology**					0.98		
Squamous	133	2.3	1.4–3.1	Reference			
All others	389	2.3	2.0–2.8	1.00 (0.82–1.23)			
**Metastases**					0.82		
Brain metastases	128	2.1	1.4–2.6	Reference			
Other metastases only	379	2.3	2.0–2.8	1.01 (0.81–1.25)			
No metastases	15	3.3	1.4–7.0	0.85 (0.49–1.48)			
**Treatment line**					0.05		0.34
2	433	2.4	2.1–2.8	Reference		Reference	
3+	89	1.9	1.4–2.6	1.27 (1.00–1.61)		1.13 (0.88-1.46)	
**Time from start of previous LoT until start of nivolumab**					0.01		<0.01
<3 months	121	1.4	1.1–2.1	Reference		Reference	
≥3–<6 months	173	2.1	1.4–2.4	0.85 (0.67–1.08)		0.78 (0.61–1.01)	
≥6–<12 months	183	2.8	2.3–3.5	0.70 (0.55–0.89)		0.65 (0.50–0.84)	
≥12 months	44	4.6	3.2–7.9	0.61 (0.42–0.88)		0.55 (0.37–0.82)	

*Characteristics at time of nivolumab start if not specified

BMI, body mass index; CI, confidence interval; ECOG PS, Eastern Cooperative Oncology Group performance score; HR, hazard ratio; LoT, line of therapy; PD-L1, programmed death-ligand 1.

Figure S1: Sensitivity analysis for OS by histology in ESME-AMLC

Overall

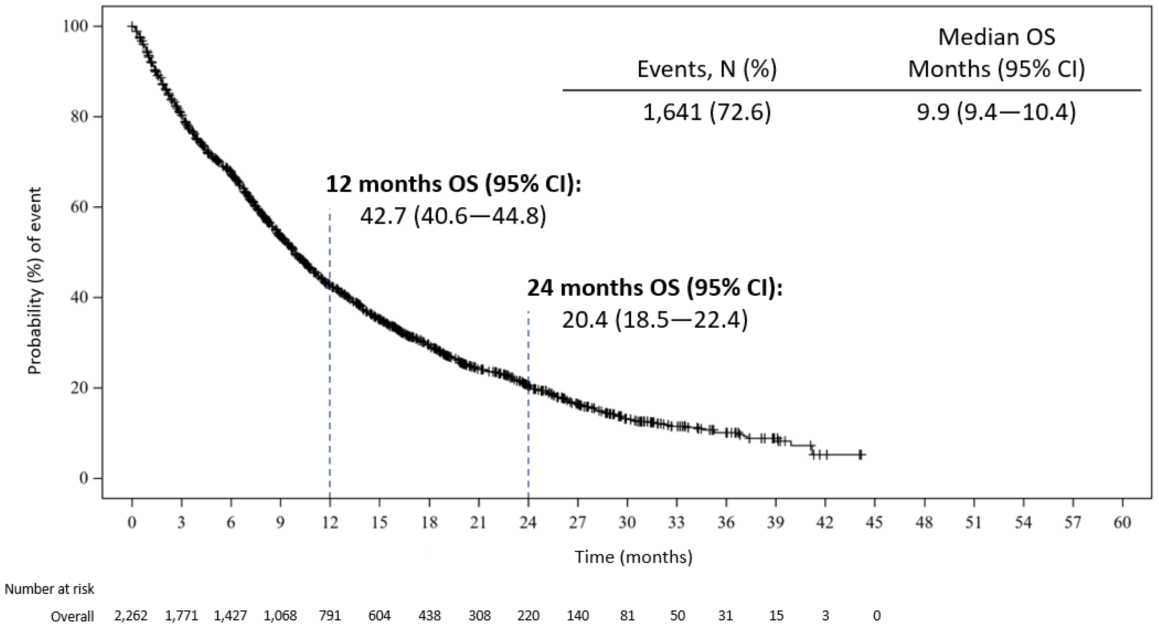

By histology

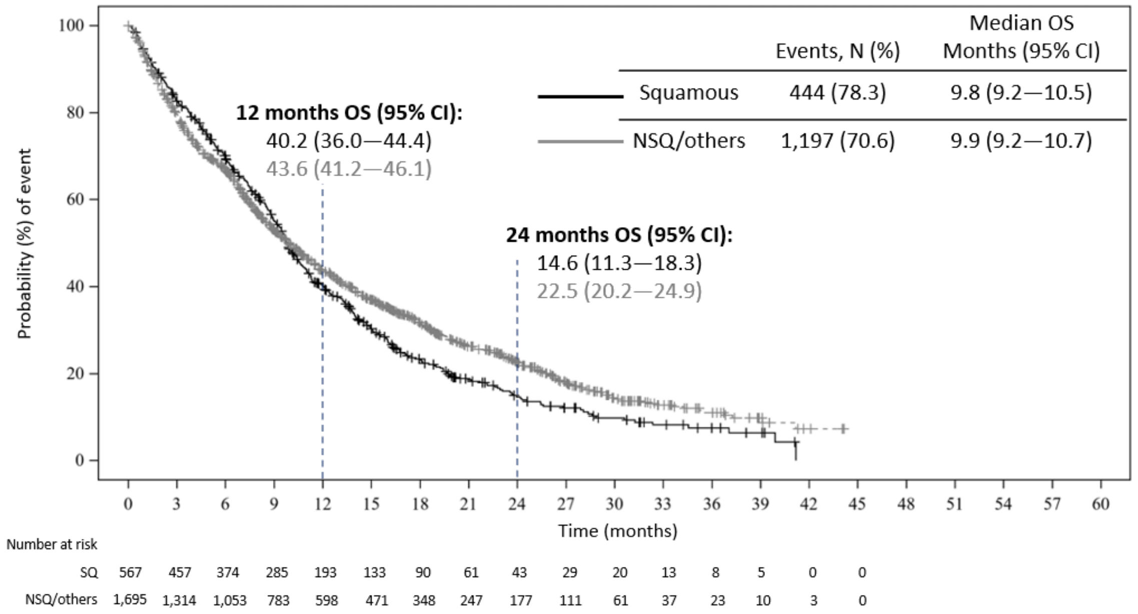

CI, confidence interval; NSQ, non-squamous; OS, overall survival; SQ, squamous.
